# Quality improvement in hepatology requires a chronic care framework

**DOI:** 10.1097/HC9.0000000000000889

**Published:** 2026-01-05

**Authors:** Chip A. Bowman

**Affiliations:** Department of Medicine, Division of Digestive and Liver Diseases, Columbia University Irving Medical Center, New York, New York, USA

Hepatology excels at managing crises. Hepatology practice involves routinely saving lives by treating acute decompensations like variceal bleeding. As highlighted in recent practice guidelines on portal hypertension, our patients’ care is being optimized by a continual process of innovation.^1^ How these innovations are implemented broadly, however, is often haphazard. Efforts to standardize and optimize care, called “quality improvement (QI),” are generally episodic, single-site, intervention-specific projects that target discrete inpatient outcomes.^2^ Most interventions address the management of crises with checklists, order sets, and educational initiatives. Though critical, they frequently occur after initial decompensation and miss opportunities earlier in the disease course. For example, hepatitis A and hepatitis B immunization can prevent severe harms, though vaccination rates among at-risk adults remain unacceptably low.^3^ This illustrates a broader challenge: even when effective preventive interventions exist, they remain isolated from the larger chronic liver disease (CLD) care pathway. This separation underscores the need for a longitudinal QI framework in hepatology.

## THE CHALLENGE

Hepatology can excel at preventing crises. By integrating QI across the whole trajectory of CLD, we can move from programs that achieve short-term gains to a program of prevention through sustainable, system-level improvement. Existing hepatology QI concepts focus on viral hepatitis immunization, fibrosis staging in metabolically at-risk persons,^4^ and inpatient decompensation management. In primary care, incorporating the Chronic Care Model (CCM) has improved outcomes and reduced healthcare costs.^5^ Applying this model to CLD has been proposed and represents an opportunity to reframe QI approaches by addressing the CCM pillars of health care organizations, delivery system design, decision support, clinical information systems, self-management support, and community resources.^6^ This gap creates an opportunity for hepatology to reimagine QI not as episodic project work, but as a longitudinal system of care aligned with the realities of chronic liver disease. A QI–CCM (Quality Improvement–Chronic Care Model) applies these pillars simultaneously and longitudinally to CLD, creating automated detection pathways, standardized clinical workflows, and closed-loop follow-up systems that prevent progression and sustain recovery. Adapting a QI–CCM approach to hepatology QI will shift the strategy from reactive to preventative.

## OPPORTUNITY

Alcohol use disorder (AUD) is the quintessential chronic disease QI problem squarely within the purview of hepatology and an exemplar of the benefit of a QI–CCM approach. This approach is a continuous, automated system that links detection directly to action. Relapse-prone and socially complex, AUD frequently leads to alcohol-associated liver disease (ALD). While it might often be the first point of contact with the medical system, waiting until an episode of alcoholic hepatitis to intervene in ALD is clinically and ethically insufficient. Screening tools, brief interventions, referral pathways, pharmacotherapy initiation, and post-transplant relapse-prevention strategies each represent distinct QI opportunities.^7^,^8^ Given that ALD is now the leading indication for transplantation,^9^ efforts to improve the quality of clinical care throughout the continuum should align with current practice guidelines. One of the recommended patient-reported outcomes in the practice metrics guidelines is the patient’s ability to avoid alcohol, which has been shown to be helped through integrated care models that combine hepatology and addiction services to reduce future decompensating events and hospitalizations.^10^,^11^

Across the timespan of ALD as a chronic disease, the QI–CCM goals remain consistent: reduce harmful drinking, prevent progression and decompensation, avoid hospitalizations, and sustain long-term recovery. To operationalize a QI–CCM approach for ALD, a stage-based implementation model can be applied. This approach embeds standardized detection workflows, automated risk-based prompts, structured linkage to addiction and medical therapy, clinical-informatics dashboards, and patient–caregiver self-management tools across the full ALD continuum from early disease through compensated and decompensated cirrhosis to the pre-transplant and post-transplant period. In early disease, delivery system design can standardize workflows with AUDIT-C screening in primary care and ED settings, while decision-support tools automatically prompt timely initiation of pharmacotherapy and psychotherapy referrals. In compensated cirrhosis, clinical-informatics dashboards help track visit adherence, abstinence, and care gaps, complemented by self-management tools that reinforce relapse-prevention strategies and ensure a closed-loop QI system. During episodes of decompensation, health care organizations can support structured pathways, such as automatic psychiatry consultation for alcoholic hepatitis and early post-discharge follow-up clinics, to reduce readmissions. Along the transplant pathway, community resources strengthen recovery through pre-transplant and post-transplant support groups, while clinical-informatics systems monitor abstinence biomarkers and follow-up adherence. Together, these CCM-aligned interventions embed QI throughout the ALD disease course.

**FIGURE 1:**
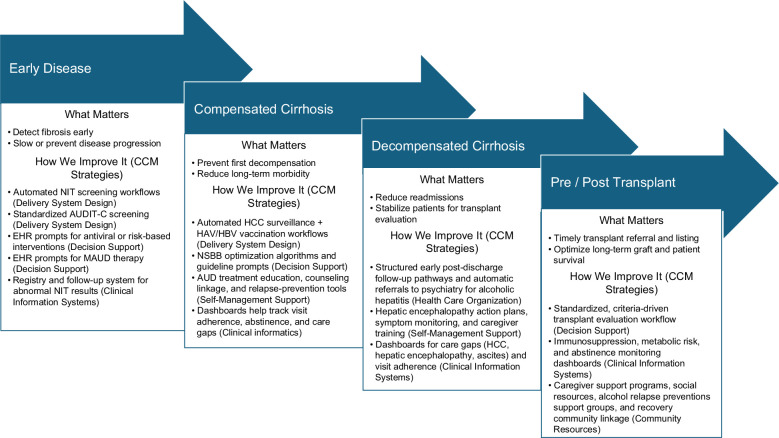
QI–CCM (Quality Improvement–Chronic Care Model) with stages of chronic liver disease identified with opportunities for care advancement that align with different pillars of the Chronic Care Model.

Reorienting hepatology QI toward prevention and continuity with a QI–CCM approach would shift the field from reacting to crises to sustaining health. Figure 1 maps CCM pillars to major stages in the CLD trajectory as QI–CCM, illustrating where structured, automated, longitudinal QI interventions can prevent progression and stabilize chronic disease. QI–CCM reorients hepatology toward prevention by ensuring that every stage of care, including early detection, compensated cirrhosis management, decompensation, and transplant, is supported by automated identification, standardized workflows, and closed-loop follow-up. The impact is clear: fewer decompensations, improved survival, reduced costs, and a more sustainable care model for a growing patient population. Adopting a QI–CCM framework would not simply refine existing practices; it would redefine hepatology as a discipline committed to proactive, preventive, and population-oriented care.
